# Reliability of a viva assessment of clinical reasoning in an Australian pre-professional osteopathy program assessed using generalizability theory

**DOI:** 10.3352/jeehp.2017.14.1

**Published:** 2017-01-20

**Authors:** Brett Vaughan, Paul Orrock, Sandra Grace

**Affiliations:** 1 School of Health and Human Sciences, Southern Cross University, Lismore, Australia; 2 College of Health and Biomedicine, Victoria University, Melbourne, Australia; 3 Institute of Sport, Exercise and Active Living, Victoria University, Melbourne, Australia; Hallym University, Korea

**Keywords:** Australia, Osteopathic medicine, Physical examination, Psychometrics, Reproducibility of results

## Abstract

Clinical reasoning is situation-dependent and case-specific; therefore, assessments incorporating different patient presentations are warranted. The present study aimed to determine the reliability of a multi-station case-based viva assessment of clinical reasoning in an Australian pre-registration osteopathy program using generalizability theory. Students (from years 4 and 5) and examiners were recruited from the osteopathy program at Southern Cross University, Lismore, Australia. The study took place on a single day in the student teaching clinic. Examiners were trained before the examination. Students were allocated to 1 of 3 rounds consisting of 5 10-minute stations in an objective structured clinical examination-style. Generalizability analysis was used to explore the reliability of the examination. Fifteen students and 5 faculty members participated in the study. The examination produced a generalizability coefficient of 0.53, with 18 stations required to achieve a generalizability coefficient of 0.80. The reliability estimations were acceptable and the psychometric findings related to the marking rubric and overall scores were acceptable; however, further work is required in examiner training and ensuring consistent case difficulty to improve the reliability of the examination.

The assessment of clinical reasoning is a challenge for health profession educators who grapple with evolving understandings of the complex nature of reasoning in practice. It appears that clinical reasoning is more closely aligned with clinical knowledge and knowledge organization than with problem-solving capacity [1]. The literature confirms that the context-specific nature of clinical reasoning requires multiple assessments in different contexts by different assessors to optimize the validity and reliability of the overall assessment. The emerging clinical reasoning literature in osteopathy includes descriptions of the reasoning processes of practicing osteopaths [2] and educators [3], and the challenges associated with clinical reasoning in osteopathy [4]. The assessment of clinical reasoning in osteopathy has also been explored [5,6]. Orrock et al. [5] developed a viva examination to assess clinical reasoning in Australasian osteopathy students. That study provided initial evidence supporting the validity of the scores derived from the examination. The authors stated that further work on the marking rubric was required, as was evaluation of the reliability of the assessment. The present study aimed to evaluate modifications to the rubric and the reliability of a clinical reasoning viva assessment in a pre-professional osteopathy program leading to registration as an osteopath in Australia.

## Study design

This was a cross-sectional study designed to evaluate the reliability of a 5 station objective structured clinical examination (OSCE)-format examination.

## Materials and/or subjects

Students enrolled in the fourth and fifth years of the osteopathy program at Southern Cross University (SCU), Lismore, Australia were invited to participate in the study. Participation was not a requirement for any academic subject in their program; however, students were provided with feedback about their performance. Examiners were recruited from the academic and clinical education staff of the SCU osteopathy program. Training was provided to the examiners in the form of a training manual, training video, and an examiner training session that lasted for 1.5 hours immediately before the viva examination.

## Technical information

Students were allocated to 1 of 3 circuits and cycled through 5 stations in an OSCE-type format. Each station lasted for 10 minutes, during which time the examiner worked through a 3-stage clinical history with the student. The study took place on a single day in the student teaching clinic on April 12, 2016. The examination process was as follows, and the content of question items is presented in Table 1: first, the student entered the room; second, the examiner presented stage 1 of the case to the student to read; third, the examiner asked Q1 and Q2 from the rubric; fourth, the examiner presented stage 2 of the case to the student to read; fifth, the examiner asked Q3, Q4, Q5, Q6, and Q7 from the rubric; sixth, the examiner presented stage 3 of the case to the student to read; and seventh, the examiner asked Q8, Q9, Q10, Q11, and Q12 from the rubric.

Each of the clinical histories was taken from the examination developed by Orrock et al. [5], and each student was marked by the examiner using a modified rubric as suggested by those authors (Appendix 1). Modifications to the rubric were guided by the correlations between multiple items observed in the study of Orrock et al. [5]. Each examiner assessed the student based on only a single clinical history scenario, and the examiner was not required to total up the marking rubric. Question 12 did not contribute to the students’ total score for the examination.

## Statistics

Descriptive statistics and reliability estimations (ordinal Cronbach alpha and McDonald omega) were generated for the examination in R ver. 3.3.0 (The R Foundation for Statistical Computing, Vienna, Austria; https://www.r-project.org/) using the ‘userfriendlyscience’ package ver. 0.4–1 (http://userfriendlyscience.com). Generalizability analysis was used to evaluate the reliability of the examination [7] using G_String IV (The Program for Educational Research and Development, Hamilton, ON, Canada; http://fhsperd.mcmaster.ca/g_string/). The generalizability (G) study had a fully crossed design with 3 facets: all ‘students’ participating in the exam were examined by all ‘examiners’ on all ‘items’ on the rubric (*student×examiner ×item*). Examiners were treated as a random facet and items were treated as a fixed facet. This design did not allow for the identification of variance due to the case and examiner, as each examiner only assessed 1 case. The examination was designed to assess the students’ clinical reasoning ability; therefore, the absolute error (Φ) was the chosen reliability coefficient [7]. A decision study was performed by changing the number of examiners/stations to investigate the number of stations required for a high-stakes assessment.

## Ethical approval

The study was approved by the Southern Cross University Human Research Ethics Committee (ECN-15-237).

Fifteen students and 5 examiners were recruited for the examination. All examiners participated in the examiner training program. The mean student score was 34.3± 7.2 out of 55 (Table 1). The Cronbach alpha value was 0.88 (95% confidence interval, 0.84 to 0.92) for the modified rubric, and removing an item from the marking rubric did not improve this value. The McDonald omega (hierarchal) was 0.71, supporting the calculation of a total score for the examination. The G-coefficient (Φ) was 0.53; that is, just over half of the variation in the results was due to differences between student performance on the examination. A generalizability coefficient of 0.80 would have been achieved for 18 examiners/stations [7]. The variance components are presented in Table 2. Residual and systematic error accounted for the largest variance, at over 37%. The raw data file is available in Supplement 1.

High-stakes assessments need to be standardized to ensure reliability, and high-stakes viva assessments have reported acceptable reliability. The present study evaluated the reliability of a clinical reasoning viva examination in an Australian pre-professional osteopathy program. The reliability estimations supported both the internal structure of the modified rubric and the calculation of a total score. The Φ-coefficient for the 5 examiners was 0.53, suggesting that 53% of the variance in the students’ total score was attributable to real differences in student performance on the examination. To achieve an acceptable coefficient for high-stakes decision-making (> 0.80), 18 examiners/stations would have been required [7]. Such a result suggests the proposed format of the examination may not be reliable without further review and re-evaluation.

The greatest variance was attributable to residual and systematic error. The *examiner* and *student×examiner* facets both contributed approximately 20% of the variance, suggesting that the examiners were a substantial contributor to a student’s score. *Examiner* variance was approximately double that of *student* variance, suggesting the mean scores given by the examiners on 1 case were more variable than the mean student score across all 5 cases. That is, little variation was found in student performance across the examination, as supported by the small percentage of variance attributable to the *student* facet. However, the study design did not allow for the influence of case difficulty/specificity to be partitioned out from the examiner facet, meaning that there may have been variability in the difficulty of each case, which was subsequently reflected in the variance resulting from the *examiner* facet. Previous work using the same cases did review the difficulty of each case and suggested that they were comparable, suggesting that the influence of the examiners may account for the result. Students were also scored differently by different examiners, as suggested by the *student×examiner* interaction. This could have been due to actual student performance, or prior knowledge of student performance. The latter is possible since the students and examiners were recruited from the same teaching program, and this may account for examiner training not being as successful as anticipated.

The *items* facet supports the Cronbach alpha and McDonald’s omega reliability estimations, but also demonstrates some variability in the item difficulty across the items on the marking rubric. That said, the items on the rubric made only a minor contribution to score variance, providing support for its use in the assessment. Further support for the rubric itself is provided by the small variance components for the *student×items* and *examiner×items* interaction terms.

The results of the present study suggest that further examiner training is required in order to improve the reliability of the examination. A number of the examiners reported difficulty completing the full suite of questions in the time allocated, and also felt that more substantial model answers would improve their grading decisions. It would also be of value to have the examiners conduct the same examination with different cases, in order to ascertain whether case specificity or examiner stringency contributed to the substantial error and the variance due to the *examiner* facet. Having 2 examiners for each case may also improve the reliability, although the potential benefit would need to be offset against the extra cost. The present study had some limitations. The small student numbers in the current study mean that our findings may not have been representative of the performance of the entire student body. There is also a possibility of self-selection bias on the part of both the students and the examiners. Students may have chosen to participate as preparation for upcoming examinations and to receive feedback. Examiner familiarity with the students is another limitation, which could be addressed by including examiners from outside the SCU teaching program. Further research into the examination is warranted following examiner training and a review of cases prior to implementation as a high-stakes assessment of clinical reasoning in osteopathy.

## Figures and Tables

**Table 1.** Descriptive statistics for the clinical reasoning viva examination rubric in an Australian pre-professional osteopathy program in 2016

Item	Mean ± standard deviation	Median (range)
Q1: How have you interpreted the given information so far?	3.4 ± 0.9	3 (1-5)
Q2: What further information is required to clarify the presenting complaint?	3.5 ±0.8	4 (1-5)
Q3: What are the primary cues and connections in the additional case information and why?	3.5 ±0.9	4 (2-5)
Q4: What are your differential diagnoses? Are there any red flags in this case?	3.3 ±0.8	3 (2-5)
Q5: Upon what literature and evidence are you basing your ideas about potential differential diagnoses, and	2.9 ± 1.0	3 (2-5)
examinations?		
Q6: What is your rationale for your choice of differential diagnoses?	3.1 ±0.8	3 (1-5)
Q7: What examination and investigations will you use to rule in/rule out differential diagnosis?	3.1 ±0.9	3 (1-5)
Q8: Can you now tell me your working diagnosis and your overall management plan?	3.1 ±0.8	3 (1-5)
Q9: If patient does not respond as expected OR incorrect working diagnosis OR your overall management plan hasn't worked, can you tell me what you would do?	2.8±0.9	3 (1-4)
Q10: What would you do if the patient was male/female /younger/older?	2.8±0.9	3 (1-4)
Q11: How have you used the osteopathic principles in your reasoning in this case?	2.8±0.9	3 (1-4)
Total	34.3 ±7.2	35 (20-50)

**Table 2.** Variance components for the generalizability study of the clinical reasoning examination in an Australian pre-professional osteopathy program in 2016

Effect	Degrees of freedom	Sum of squares	Variance component	Percentage of variance component (%)
Student	14	99.08	0.08	9.54
Examiner	4	118.81	0.16	18.43
Items	10	57.62	0.06	7.25
Student × examiner	56	132.59	0.19	21.01
Student × items	140	58.48	0.02	1.96
Examiner × items	40	35.42	0.04	4.19
Student × examiner × items^a)^	560	185.56	0.33	37.61

a) Residual and systematic error.

## Supplementary materials

Supplement 1. Data file is available from http://doi.org/10.6084/m9.figshare.4491107

Supplement 2. Audio recording of the abstract.

## Appendix 1.

**Table:** Marking rubric for the viva assessment of clinical reasoning in an Australian pre-registration osteopathy program modified by the present authors

Attribute/descriptor	Questions	1	2	3	4	5
Provide presenting complaint details						
Analysis: demonstrates interpretation of case information						
	Q1: How have you interpreted the given information so far?	Poor/no attempt to interpret information from case.	Limited interpretation of case information	Interprets information from case at an acceptable level.	Thorough interpret information from case.	Comprehensive interpretation of information from case.
	Q2: What further information is required to clarify the presenting complaint?	Poor/no attempt to synthesise relevant information from case.	Limited attempt to synthesise relevant information from case.	Sound attempt to synthesise relevant information from case.	Thorough synthesis of relevant information from case.	Comprehensive synthesis of relevant information from case.
Provide additional presenting complaint details						
Heuristics: makes connections between cues in the case and includes the patients’ context in the additional information.						
	Q3: What are the primary cues and connections in the additional case information and why?	Unable to identify major cues and make connections.	Identifies a limited number of cues and make connections between them.	Identifies main cues and make connections between them.	Identifies majority of cues and make connections between them.	Identifies all relevant cues and make connections between them.
Inference and information processing: uses knowledge to generate ideas about differential diagnosis and treatment.						
	Q4: What are your differential diagnoses? Are there any red flags in this case?	Includes irrelevant differential diagnoses. Omits red flags present in the case.	Includes a limited number of relevant and unlikely differential diagnoses. Omits red flags present in the case.	Identifies relevant differential diagnoses. Includes red flags present in the case.	Identifies most likely differential diagnosis and other relevant differential diagnoses. Includes red flags present in the case.	Orders relevant differential diagnoses from most to least likely. Includes red flags present in the case.
	Q5: Upon what literature and evidence are you basing your ideas about potential differential diagnoses, and examinations.	Poor application of knowledge with use of irrelevant literature.	Limited application of knowledge with limited use of relevant literature.	Appropriate application of knowledge and use of relevant literature.	Thorough application o knowledge with use of relevant literature.	Comprehensive application of knowledge with use of relevant literature.
Logic: provides a sound rationale for differential diagnoses and choice of examinations.						
	Q6: What is your rationale for your choice of differential diagnoses?	Unable to provide sound reasoning for choice of differential diagnoses.	Limited use of reasoning for choice of differential diagnoses.	Provides sound reasoning for choice of differential diagnoses.	Provides thorough reasoning for choice of differential diagnoses.	Comprehensive reasoning for choice of differential diagnoses.
	Q7: What examination and investigations will you use to rule in/rule out differential diagnosis?	No clear strategy for ruling in/ruling out differential diagnosis.	Limited use of strategy for ruling in/ruling out differential diagnosis.	Sound strategy for ruling in/ruling out differential diagnosis.	Thorough strategy for ruling in/ruling out differential diagnosis.	Comprehensive strategy for ruling in/ruling out differential diagnosis.
Provide examination findings						
C ognition: thinks aloud about choices of differential diagnosis, examination, overall Management Plan, ability to adapt to emerging information OR ancillary question						
	Q8: Can you now tell me your working diagnosis and your overall management plan?	Working diagnosis not consistent with history and examination findings. Overall management inappropriate for working diagnosis.	Working diagnosis not consistent with history and examination findings. Aspects of overall management appropriate for working diagnosis.	Working diagnosis consistent with history and examination findings. Overall management appropriate for working diagnosis.	Thorough working diagnosis consistent with history and examination findings. Thorough rationale for overall patient management.	Comprehensive working diagnosis consistent with history and examination findings. Overall management addresses multiple aspects of the patient's presentation.
	Q9: If Patient does not respond as expected OR incorrect working diagnosis OR your overall management plan hasn't worked, can you tell me what you would do?	Poor/no attempt to reason alternative options with this case.	Limited ability to reason aloud alternative options with this case.	Reasons aloud through problem solving strategies and alternative options with this case.	Reasons aloud problem solving strategies in relation to alternative options with this case.	Comprehensively articulates alternative options with this case.
	Q10: What would you do if patient was male/female /younger/older?	Is not able to articulate alternative options	Limited skills in articulating alternative options	Illustrates ability to articulate reasonable alternative options	Thoroughly demonstrates ability to articulate their reasoning and decision(s) in accordance with new information	Comprehensively demonstrates flexibility in reasoning, with ability to adjust differential diagnoses and treatment plans according to new information.
Meta-cognition: demonstrates ability to reflect on clinical reasoning process, including osteopathic principles.						
	Q11: How have you used the osteopathic principles in your reasoning in this case?	Poor/no attempt at reflection on osteopathic principles.	Limited attempt to reflect on strengths and weaknesses in clinical reasoning.	Sound attempt to reflect on strengths and weaknesses in clinical reasoning.	Thorough reflections on strengths and weaknesses in clinical reasoning.	Comprehensively reflects on strengths and weaknesses in clinical reasoning.
	Q12: What are your thoughts about how you handled this case? What would you improve on in your handling of this case?	Ungraded				
